# Metabonomic Investigation of Single and Multiple Strain *Trypanosoma brucei brucei* Infections

**DOI:** 10.4269/ajtmh.2011.10-0402

**Published:** 2011-01-05

**Authors:** Jia V. Li, Jasmina Saric, Yulan Wang, Jürg Utzinger, Elaine Holmes, Oliver Balmer

**Affiliations:** Biomolecular Medicine, Department of Surgery and Cancer, Faculty of Medicine, Imperial College London, London, United Kingdom; State Key Laboratory of Magnetic Resonance and Atomic and Molecular Physics, Wuhan Centre for Magnetic Resonance, Wuhan Institute of Physics and Mathematics, Chinese Academy of Sciences, Wuhan, People's Republic of China; Department of Epidemiology and Public Health, Swiss Tropical and Public Health Institute, Basel, Switzerland; University of Basel, Basel, Switzerland; Department of Medical Parasitology and Infection Biology, Swiss Tropical and Public Health Institute, Basel, Switzerland; Department of Ecology and Evolutionary Biology, Yale University, New Haven, Connecticut; Institute of Zoology, University of Basel, Basel, Switzerland

## Abstract

Although co-infections are common and can have important epidemiologic and evolutionary consequences, studies exploring biochemical effects of multiple-strain infections remain scarce. We studied metabolic responses of NMRI mice to *Trypanosoma brucei brucei* single (STIB777AE-Green1 or STIB246BA-Red1) and co-infections using a ^1^H nuclear magnetic resonance (NMR) spectroscopy-based metabolic profiling strategy. All *T. b. brucei* infections caused an alteration in urinary biochemical composition by day 4 postinfection, characterized by increased concentrations of 2-oxoisocaproate, D-3-hydroxybutyrate, lactate, 4-hydroxyphenylacetate, phenylpyruvate, and 4-hydroxyphenylpyruvate, and decreased levels of hippurate. Although there were no marked differences in metabolic signatures observed in the mouse infected with a single or dual strain of *T. b. brucei*, there was a slower metabolic response in mice infected with *T. b. brucei* green strain compared with mice infected with either the red strain or both strains concurrently. Pyruvate, phenylpyruvate, and hippurate were correlated with parasitemia, which might be useful in monitoring responses to therapeutic interventions.

## Introduction

Parasitic infections are often considered as homogeneous entities, but there is growing evidence that infections of most parasite species commonly consist of multiple genetically distinct strains.^1^–^5^ This diversity can have profound effects on infection and disease progression, transmission dynamics, and clinical patient management. For example, different strains of the protozoan blood parasite *Trypanosoma brucei* can inhibit each other, resulting in lower parasitemia and enhanced host survival in individuals co-infected with two strains.^6^ In *Plasmodium falciparum*, increased multiplicity of infection can increase or decrease the risk of clinical malaria, depending on host age and disease prevalence.^7^ Multiple strain infections can thus benefit the host in certain circumstances. Moreover, multiple strain infections are important to recognize in clinical settings in cases where strains respond differently to treatment or other interventions. For instance, treatment regimes may fail if they are directed only toward the dominant strain, with concurrent strains not being recognised and targeted.^8^,^9^

*Trypanosoma brucei*, transmitted by the bite of the tsetse fly (*Glossina* spp.) vector,^10^,^11^ causes invariably lethal human African trypanosomiasis (sleeping sickness) and animal Nagana in remote rural parts of sub-Saharan Africa. Human disease is caused by two sub-species, *Trypanosoma brucei rhodesiense* (Eastern and Southern Africa, acute disease course) and *Trypanosoma brucei gambiense* (Western and Central Africa, chronic disease progression), whereas *T. b. brucei* is non-human infective.^12^,^13^ Different strains of *T. brucei* exhibit considerable variability. A study in Uganda showed for instance genetic differences in *T. b. rhodesiense* infections between two different rural villages (e.g., Tororo and Soroti). These intra-parasite variations are reflected in the patient immune response and consequently in disease progression.^14^ In view of multiple strain infections being common in *T. brucei*,^1^ it is important to further our understanding of hosts co-infected with different strains of *T. brucei*.

Nuclear magnetic resonance (NMR) spectroscopy-based metabolic profiling has emerged as a powerful approach to investigate mechanisms of host-parasite interaction and shows promise for early disease diagnosis.^15^ The multivariate structure of NMR spectral data requires appropriate statistical analysis to elucidate and interpret complex datasets with typically more variables than samples.^16^ Widely used methods include unsupervised principal component analysis (PCA), which provides an overview of the intrinsic similarities and differences in a dataset revealing groupings, outliers and time trajectories, and supervised orthogonal signal corrected-projection to latent structure-discriminant analysis (O-PLS-DA).^17^ The latter method is particularly suitable for identifying systematic changes relating to distinct classes (e.g., infected versus non-infected control groups).^18^ A series of host-parasite interactions have been investigated at the molecular level, both for helminths and protozoan.^19^ However, although multiparasitism is the norm rather than the exception in developing country settings,^20^,^21^ little attention has been paid to interactions between multiple strains of a single parasite or different species of parasites co-infecting a single host. Recently, ^1^H NMR spectroscopy-based metabolic profiling has been used to characterize the global responses in the hamster to two helminth species.^22^ To our knowledge, differences among multiple strains of a single parasite species have yet to be investigated in a systematic manner. Recently, the metabolic profile of single strain *T. b. brucei* infections in mice has been studied and a broad range of metabolic changes involved in energy metabolism, symbiotic microbiota-related metabolism, and inflammatory response were found.^23^ Here, we take our previous study a step further. We used two different *T. b. brucei* strains and determined whether they cause different metabolic responses in the same host and whether the metabolic profiles of multiple strain infections differ from single infections to discern the interactions between the different strains.

## Materials and methods

### Study approval, parasite strains, and mice.

The study was approved by the veterinary department of Basel-Stadt, Switzerland (permit no. 2073). Two *T. b. brucei* strains, STIB246BA-R1 (subsequently called “*red*”) and STIB777AE-G1 (subsequently called “*green*”), transfected with a red and green fluorescent protein gene, respectively, to enable visual distinction for individual population density tracking,^24^ were used to infect 33-day-old outbred female NMRI mice (*Mus musculus*; purchased from RCC Ltd., Itingen, Switzerland). The two strains derive from independent isolations (from a kongoni [*Alcelaphus buselaphus*] in Tanzania and a tsetse fly [*Glossina fuscipes*] in Uganda, respectively) and are distinct in nuclear *COI* sequence and microsatellite markers.^25^

This study is a further analysis of the “equal virulence experiment” presented elsewhere^6^ with *green* corresponding to strain *green*_VIR_ in that study. Strain *green*_VIR_ was preferred over *green*_AVIR_^6^ because it exhibits identical growth characteristics to *red*, so that different metabolic responses can be attributed to the strain (genotype) rather than to its growth characteristics (phenotype). Mice were kept in cages with a maximum of five animals per cage and provided food and water *ad libitum*. All mice in one cage belonged to different experimental treatments to avoid systematic cage effects.

### Experimental design and infections.

Five mice each were infected intraperitoneally with 300 μL of saline solution containing 1) 10^7^ parasites of *red* (“red treatment,” R_7_); 2) 10^7^ parasites of *green* (“green treatment,” G_7_); 3) 10^7^ parasites of *red* and 10^5^ parasites of *green* (“mixed treatment,” R_7_G_5_); 4) 10^7^ parasites each of *red* and *green* (“mixed treatment,” R_7_G_7_); or 5) no parasites (“uninfected control”).

### Sample collection, measurements, and statistical analysis.

Measurements were taken daily until day 4 postinfection. Parasite density (parasitemia, expressed as parasites per mL host blood), mouse weight (g), erythrocyte (red blood cell), and thrombocyte concentrations (per mL host blood) were measured. Parasite and blood cell counts were determined by a Becton Dickinson FACScan flow cytometer (Becton, Dickinson and Company, Franklin Lanes, NJ) using the first drop of tail blood as described elsewhere.^24^ Apart from the metabolic analysis (see below), statistical analyses were performed using R version 2.8.1^26^ with details provided elsewhere.^6^

Urine and fecal samples for metabolic analyses were collected between 06:00 and 08:00 hours, followed by mouse weight and blood measurements, to minimize metabolic variation caused by diurnal fluctuation, 1 day before the infection and at days 1, 3, and 4 postinfection. Although the “equal virulence experiment” was continued until host death,^6^ sample collection for the current metabolic profiling study was halted at 4 days postinfection, because several animals were already moribund and thus loss of the more subtle strain-related variation would be expected. We aimed at collecting a minimum of 30-μL urine per mouse at each sampling time point, which was difficult for some individuals because of general ill health and potential dehydration. We did not separate mice into individual cages to generate more biofluid samples to avoid stress-related changes. Samples were kept at −40°C before NMR-based metabolic profiling.

### ^1^H NMR spectroscopy and multivariate data analysis.

For each urine sample, 30 μL of urine were mixed with 25 μL of sodium phosphate buffer (pH 7.4) containing 0.01% sodium 3-(trimethylsilyl) propionate-2,2,3,3-d_4_ (TSP) for spectrum calibration, 10% H_2_O and 90% D_2_O for field lock of spectrometer, and 3 mM Na-azide for eliminating eventual bacterial contamination, was transferred into a capillary NMR tube with an outer diameter of 1.7 mm for NMR analysis. For each fecal sample, two fecal pellets (~0.06 g) were transferred into a 1.5 mL Eppendorf tube (Natick, MA) containing 750 μL of phosphate buffer as in urine sample preparation, homogenized and sonicated for 30 min. Approximately 600 μL of supernatant was transferred into an NMR tube with an outer diameter of 5 mm for NMR analysis following centrifugation at 10,000 *g* for 10 min. All samples were analyzed on a Bruker 600 MHz spectrometer (Bruker; Rheinstetten, Germany), operating at 600.13 MHz at a temperature of 27°C. A standard pulse sequence (recycle delay [RD]-90°-*t*_1_-90°-*t*_m_-90°-acquisition) was used to acquire one-dimensional (1-D) spectra. The saturation of the water signal was achieved during the mixing time (*t*_m_) of 100 ms and RD of 2 s. The 90° pulse was adjusted to ~10 μs and *t*_1_ was set to 3 μs. A total of 256 scans were accumulated into 32,768 data points with a spectral width of 20 ppm. For the purpose of metabolite identification, two-dimensional (2-D) spectra were also acquired from selected samples based on our previously published parameters.^15^

Phased, baseline corrected, and calibrated spectra were imported into MATLAB software (MathWorks, version 7.8.0.347) for multivariate data analyses. The spectral region containing the water resonance (δ 4.6–5.0) was removed to minimize the distortion effect of the water peak on the baseline and the regions between δ 5.3 and 6.3 were also omitted because partial cross saturation of the urea signal at this region would bias the analysis.^16^ Normalization to the total area of the remaining spectrum was applied to each spectrum before conducting multivariate data analyses.

The PCA with unit variance scaling^17^ and O-PLS-DA^18^ were used for multivariate data analyses. The PCA generates scores plots, which allow clusters and structures within a dataset to be visualized, and loadings plots that indicate which variables contribute the highest weighting to the distribution of the samples in the scores plot. In the O-PLS-DA algorithm, the *X* matrix comprised observations (NMR spectra) and the *Y* matrix contained class information (e.g., infection status). There was evidence of strain-related metabolic differences in the PCA scores plots, and hence O-PLS-DA models were constructed on the basis of unit variance scaled NMR spectral data with one PLS component to discriminate two infection statuses (e.g., uninfected control versus infected; single infection versus multiple strain infection), and one orthogonal component that removes systematic variation unrelated to the class discrimination. Because the number of animals per group was relatively small (*N* = 5; caused by adherence to the 3R rule: reduce, refine, and replace), four quantitative metrics were used to assess the quality of each model. R^2^*X* describes the goodness of fit of a model and represents the proportion of variation in *X* explained by the model. The predictivity of a model, Q^2^*Y*, measures how well the *Y* matrix can be predicted. Sensitivity is defined as *tp*/(*tp* + *fn*) and specificity as *tn*/(*fp* + *tn*), where *tp* stands for true positive (an observation or a sample from a group that is correctly classified as that group) and *tn* stands for true negative (a sample not from a specific group that is correctly classified as a non-member of this specific group). The *fp* and *fn* stand for false positive and false negative, respectively (incorrect membership classifications).^27^

For the four most significantly altered urinary metabolites following experimental infection, NMR signal integration was performed to establish whether these metabolites showed a quantitative relationship with parasitemia levels. Integrals were calculated from minimally overlapped peak areas for each metabolite (δ 2.373–2.388 for pyruvate, δ 4.015–4.029 for 4-hydroxyphenylpyrvate, δ 7.401–7.448 for phenylpyruvate, and δ 7.813–7.868 for hippurate), and the resulting values taken as the relative concentration of each metabolite in the spectra.

## Results

### Host fitness, parasitemia, and blood values.

Host survival time after infection and erythrocyte and thrombocyte counts did not differ between the four infected groups at any day postinfection but were significantly different from uninfected control mice on day 4 (all *P* < 0.05). All measures significantly changed over time in infected, but not in uninfected control mice (Table 1). The two strains were both suppressed in the two mixed treatments (R_7_G_5_ and R_7_G_7_) such that the combined total parasitemia per host reached the same density as each strain alone in single infections.

### 1-D ^1^H NMR spectroscopic analysis of fecal water extract.

There were no significant changes in spectral data obtained from fecal water extracts between uninfected control and *T. b. brucei*-infected mice of any infection treatment, corroborating previous findings in mice experimentally infected with the intestinal fluke Echinostoma caproni^26^ and *Plasmodium berghei*,^28^ and underscoring that fecal water extracts are less suitable than other biological samples, e.g., uring or plasma.^29^

### 1-D ^1^H NMR spectroscopic analysis of urine.

**Figure 1.**
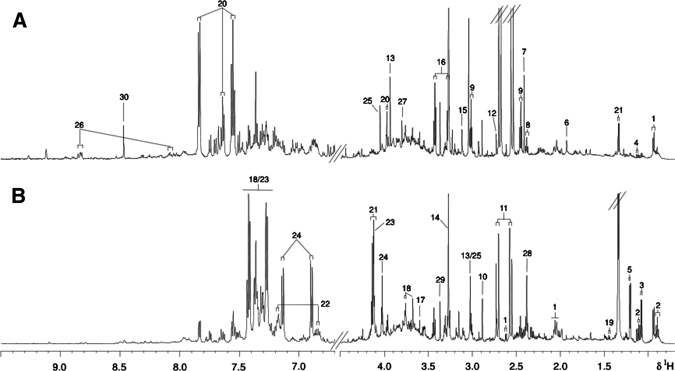
Typical 600 MHz high-resolution ^1^H nuclear magnetic resonance (NMR) urinary spectra from (**A**) an uninfected control mouse and (**B**) a mouse with a 4-day-old co-infection of *Trypanosoma brucei brucei* strains STIB246BA-R1 (*red*) and STIB777AE-G1 (*green*). Key: 1, 2-oxoisocaproate; 2, 3-methyl-2-oxovalerate; 3, 3-carboxyl-2-methyl-3-oxopropanamine*; 4, 2-oxoisovalerate; 5, D-3-hydroxybutyrate; 6, acetate; 7, succinate; 8, 3-aminopropionic acid; 9, 2-oxoglutarate; 10, trimethylamine; 11, citrate; 12, dimethylamine; 13, creatine; 14, trimethylamine *N*-oxide; 15, malonate; 16, taurine; 17, glycine; 18, phenylacetylglycine; 19, alanine; 20, hippurate; 21, lactate; 22, 4-hydroxyphenylacetic acid; 23, phenylpyruvate; 24, 4-hydroxyphenylpyruvate; 25, creatinine; 26, 1-methylnicotinamide; 27, guanidoacetate*; 28, pyruvate; 29, methanol; 30, formate. * Tentative assignment based on chemical shift comparison with literature values.

All infected mice showed similar ^1^H NMR spectral composition in urine at day 4 postinfection. However, the urinary metabolic profiles obtained from infected mice were markedly different from those of uninfected control mice, particularly in the aromatic regions, where levels of 4-hydroxyphenylpyruvate, phenylpyruvate, and 4-hydroxyphenylacetate were elevated, and hippurate was depleted among infected mice (Figure 1). Levels of lactate and D-3-hydroxybutyrate were also increased. A doublet at δ 1.08, which correlated with peaks at δ 2.50, δ 3.54, and δ 3.70 observed in a ^1^H-^1^H 2-D NMR total correlation spectroscopy spectrum, was provisionally assigned as 3-carboxy-2-methyl-3-oxopropanamine in accordance with previous investigations in the *T. b. brucei*-mouse model.^23^

### PCA of ^1^H NMR urinary spectra.

**Figure 2.**
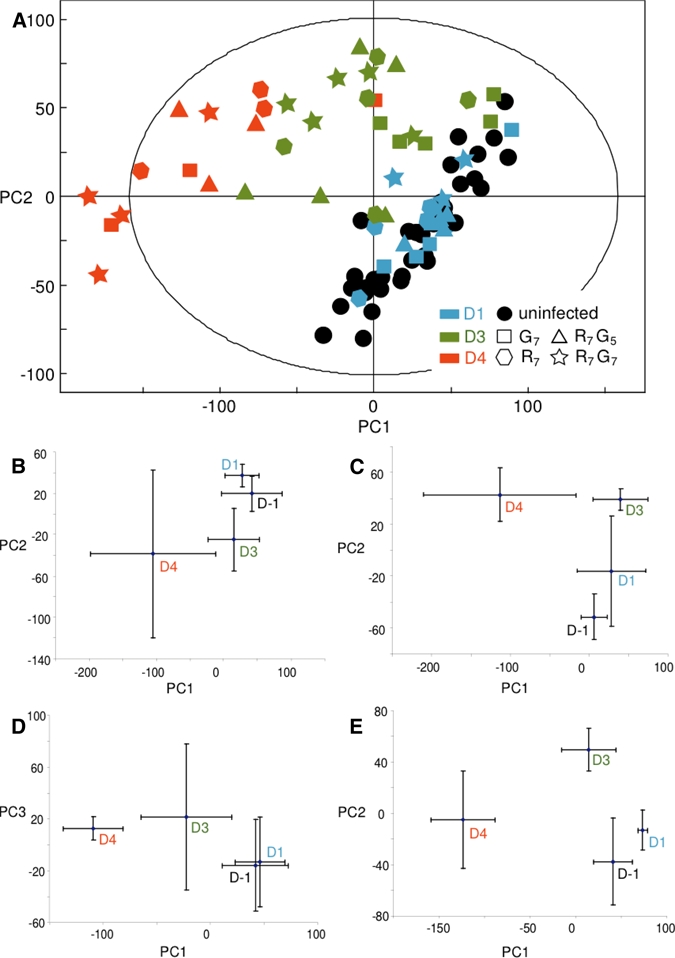
Principal component analysis (PCA) trajectory scores plots. (**A**) Plot derived from urinary nuclear magnetic resonance (NMR) spectra of all mice 1 day preinfection (black) and on days 1 (no fill), 3 (grey), and 4 (black radial) postinfection (G_7_; “square,” R_7_; “hexagon,” R_7_G_5_; “triangle,” R_7_G_7_; “star”). The group of uninfected mice (black) contains the measurements of all treatments before infection and measurements of uninfected control mice on days 1, 3, and 4 in this graph. The first two principal components (PC1 and PC2) explain 26.3% and 10.6% of total variance in the data, respectively. Sub-level PCA time trajectory plots derived from treatments (**B**) R_7_, (**C**) G_7_, (**D**) R_7_G_5_, and (**E**) R_7_G_7_ showing differentiation of preinfection (D_−1_) and postinfection at each time point (D_1_, D_3_, and D_4_). See Table 2 for treatment details. This figure appears in color at www.ajtmh.org.

The PCA analysis of the urinary spectra obtained from all mice at all time points was performed and nine outliers were observed, six of which had poor quality ^1^H NMR spectra caused by an insufficient quantity of urine (< 30 μL) for NMR analysis. The remaining three samples obtained at day 4 postinfection mapped outside the Hotelling 95% confidence ellipse caused by much higher concentrations of lactate and pyruvate (4- and 1.75-folds greater than the mean of other infected animals at day 4, respectively). These samples exerted a strong leverage on the model such that strain-related variation was obscured. Therefore, the PCA model was recalculated after removal of these samples to uncover the systematic metabolic variation related to parasitic strain effect. In the resulting PCA scores plot, animals clustered according to the progression of infection with a clear time-related trajectory along the first principal component (Figure 2A). Uninfected control mice, the preinfection time point (D_−1_) and early stage infection time point (day 1 postinfection, D_1_) grouped together as shown in Figure 2A and were separated from later stage infection time points (D_3_ and D_4_). On day 3, mice singly infected with *green* were closer to the cluster of control and early stage of infection than mice with other infection treatments. According to the loadings plot (not shown), mice in the late stage of infection were clearly distinguished from control mice and mice at early stages of infection by higher concentrations of phenylpyruvate and 4-hydroxyphenylpyruvate.

Sub-level PCA time trajectory plots of each infection treatment are displayed in Figure 2B–E, which were calculated on the basis of the average of scores from all mice at each time point with the same infection treatment. These sub-level PCA time trajectory plots further illustrate clearly time and disease progression-dependent patterns with significant metabolic deviations, such as pre- and early infection time point versus day 4 postinfection in all four cases, while the sub-level PCA trajectory plot derived from the control mice (not shown) exhibits substantial standard deviations for each time point, and hence no significant temporal movement.

### O-PLS-DA on ^1^H NMR urinary spectra.

The O-PLS-DA models were constructed to discriminate mice in the different infection treatments from uninfected controls at various time points. The predictivity values (Q^2^*Y*) of each O-PLS-DA model and the correlation coefficients of differentiating metabolites are summarized in Table 2. Given the sample size of five animals in each treatment, a correlation coefficient above 0.75 was considered significant (*P* < 0.05).^30^ On day 1 postinfection, no difference was observed between control and any type of infection. The predictivity measure (Q^2^*Y*) of the O-PLS-DA model for classifying into *red* and control mice at day 3 postinfection was 57%, whereas the predictivity value derived from *green* and control mice was only 25%. The *red*-control model showed similar sensitivity as the *green*-control model (75% versus 78%) but much higher specificity (70% versus 8%). As the infection progressed to day 4, the urinary metabolic profiles of all infection treatments converged. The metabolites 3-methyl-2-oxovalerate, D-3-hydroxybutyrate, 3-carboxy-2-methyl-3-oxopropanamine, 2-oxoisovalerate, lactate, and pyruvate were found to change the relative concentration significantly upon co-infection (Table 2). However, these metabolites did not show significant changes in O-PLS-DA models derived either from each single infection versus control or from each single infection versus co-infection. The reason is probably that these metabolites showed the same trend of changes in the single strain-infected mice as in the co-infected mice, but just failed to reach the significance level of *P* < 0.05 in the single infection models. This observation points to a qualitatively identical but quantitatively different concentrations of metabolites in multiple strain infections. The metabolic response was strikingly different from that of *P. berghei*-infected mice, which for example exhibited increased pipecolic acid as a unique biomarker (Table 2).^29^

**Figure 3.**
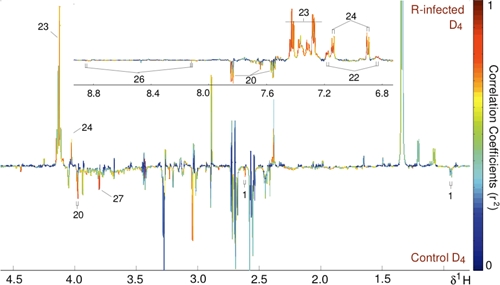
Orthogonal signal corrected-projection to latent structure-discriminant analysis (O-PLS-DA) coefficient plot derived from ^1^H nuclear magnetic resonance (NMR) spectra of urine obtained from four *red* strain-infected mice and five control mice at day 4 postinfection. Peaks pointing up show higher concentrations of metabolites in the infected group, peaks pointing down lower concentrations. The significance of contribution of each peak increases from blue to red. Q^2^*Y* = 0.85. Key to metabolite identification as in Figure 1. This figure appears in color at www.ajtmh.org.

An O-PLS-DA model of control and *red*-infected mice at day 4 is represented in Figure 3. Increased urinary concentrations of 2-oxoisocaproate, D-3-hydroxybutyrate, 3-carboxyl-2-methyl-3-oxopropanamine, pyruvate, lactate, 4-hydroxyphenylacetate, phenylpyruvate, and 4-hydroxyphenylpyruvate, and decreased levels of hippurate, guanidoacetate, and 1-methylnicotinamide were found in the *red*-infected mice compared with uninfected control mice.

### Correlation between urinary metabolites, parasitemia, and blood values.

**Figure 4.**
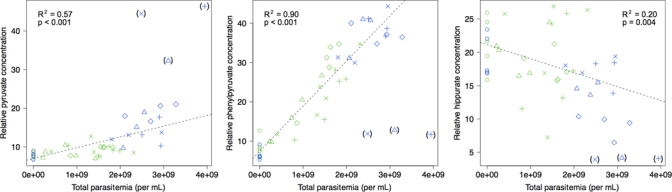
Correlation between total parasitemia (sum of *T. b. brucei* strains STIB246BA-R1 and STIB777AE-G1) per mouse and relative concentrations (in arbitrary units) of three metabolites found to be significantly altered on days 3 and 4 postinfection coded by day postinfection (green, day 3; blue, day 4) and treatment (“o,” control; “Δ,” G_7_; “+,” R_7_; “x,” R_7_G_5_; “⋄,” R_7_G_7_; see Table 2 for treatment details). The three data points in parentheses are outliers and were not included in the regression analysis. The dashed line is the linear regression line. This figure appears in color at www.ajtmh.org.

The relative concentrations of pyruvate and phenylpyruvate were strongly positively correlated with parasitemia, whereas hippurate showed a weak but statistically significant negative correlation (Figure 4), that is, urinary hippurate concentration is lower in the infected animals. Three mice from three different treatments and three different cages were excluded from the regression analysis because they were clear outliers that exhibited strikingly different behavior in the measured metabolites (but not in survival and weight on day 4) from all other data points.

## Discussion

Mice infected with *T. b. brucei* strains STIB246BA-R1 (“*red*”), STIB777AE-G1 (“*green*”), or co-infected with both strains revealed similar urinary metabolic signatures, reflecting perturbation in host energy metabolism (e.g., lactate, pyruvate, and D-3-hydroxybutyrate) and gut microbial metabolism (e.g., hippurate and 4-hydroxyphenylacetate). Animals infected only with *green* showed a slower metabolic response than those infected with either *red* alone or both strains concurrently. These findings suggest that *red* is more virulent. Those mice infected with the heavier mixed strain load showed a relatively greater number of metabolic perturbations than mice from the other groups. The same trend was confirmed in the time trajectory comparison between the infected groups, showing that the high mixed titer was the only one that induced clear separation between all time points assessed (e.g., preinfection and day 1 postinfection), indicating a stronger pathology-driven temporal variation. Although the metabolic responses to each of the infections were similar, there were many inconsistencies with the previously reported metabolic signature of infection with GVR35, another *T. b. brucei* strain maintained in our laboratories for many years.^23^ Similar responses were observed in gut microbial metabolites and organic acids, but increased excretion of pyruvate, phenylpyruvate, and 4-hydroxyphenylpyruvate was one of the key discriminatory features in the current series of infections, although it was not observed by Wang and colleagues.^23^ However, *T. b. brucei* strain GVR 35 caused a chronic infection with low parasitemia, whereas the infections in this study were acute with very high levels of parasitemia reached within a few days. Hence, the differences in the experimental protocols could account for the variation in metabolic response between the two studies. In addition, the infections in the current study were highly intensive and five animals in each group were sufficient to produce strong statistical models. Therefore, fewer animals were used than in our previous parasite-host models, which are also justified on ethical grounds (i.e., 3R rule).

The two trypanosome strains used in the current experiment reached basically identical total parasitemia levels in single and multiple infections, i.e., both strains were suppressed in the mixed infections. We have previously shown that both strains are equally suppressed but that suppression is not just the cause of the total parasite density, because in a double infection with a more slowly growing variant of the *green* strain used here, suppression of the co-infecting *red* strain was just as severe, despite much lower total parasitemia.^6^ The results of this study do not further elucidate the mechanism, by which this apparently strain-specific suppression occurs, because both strains exhibited virtually indistinguishable metabolic profiles and similar profiles were found between single and multiple strain infections.

A prominent finding was that the elevated levels of keto-acids (phenylpyruvate and 4-hydroxyphenylpyruvate), indicating alterations in the phenylalanine and tyrosine metabolism, which were also observed in *T. b. gambiense*^31^ and in *T. evansi*,^32^ were correlated with parasitemia. In the study by El Sawalhy and colleagues,^32^ the levels of phenylpyruvate and 4-hydroxyphenylpyruvate returned to normal after therapeutic intervention. Pyruvate is known to be the primary metabolite excreted by *T. brucei*.^33^,^34^ The increased level of pyruvate in the host blood may thus simply be the consequence of higher parasite density, an interpretation supported by the close correlation between parasitemia and pyruvate levels reported here. However, phenylpyruvate and 4-hydroxyphenylpyruvate are also metabolized from phenylalanine and tyrosine, respectively, by L-amino oxidase. L-amino oxidase from the venom glands of *Bothrops moojeni* and *Bothrops jararacussu* have been reported to exhibit antiviral (against Dengue virus) and antiprotozoal (against *Trypanosoma* and *Leishmania* species) activities.^35^,^36^ The elevated levels of phenylpyruvate and 4-hydroxyphenylpyruvate could also be caused by an increased L-amino oxidase activity in *T. b. brucei*-infected mice. Future studies should assess the activity of this enzyme *in vivo*. The 4-hydroxyphenylacetate is derived from 4-hydroxyphenylpyruvate by 4-hydroxyphenylpyruvate oxidase and the increased concentration of this metabolite was consistent with the higher level of 4-hydroxyphenylpyruvate. Although it is not clear to what extent these further metabolites are produced by parasite or host metabolism, the increase of these metabolites is the most marked feature in *T. b. brucei*-infected hosts.

The 1-methylnicotinamide, which was significantly decreased in infected mice, is involved in the nicotinate metabolism and is mainly produced from nicotinamide by nicotinamide *N*-methyltransferase (NNMT) in the liver. This metabolite has previously been reported to be associated with anti-thrombotic activity^37^ and may explain why infected mice consistently exhibited drastic thrombocytopenia as the first sign of disease. The 2-oxoisocaproate, which is derived from leucine, was found to be present in lower concentrations in all infected animals at day 4. This observation is in agreement with a decreased plasma level of leucine in the *T. b. brucei*-infected mice reported previously.^23^

In conclusion, this study has shown that two different strains of *T. b. brucei* resulted in similar metabolic profiles in the mouse, but there were a number of minor, yet significant, quantitative differences. We speculate that the metabolic differences between single and multiple strain infections observed at different time points are likely attributable to the infection intensity and disease progression. As far as we know, this is the first study comparing the metabolic signature of multiple strains of a single parasite species in a rodent model. We only found subtle strain differences, which might be partially explained by the small number of mice in each group (*N* = 5). However, we would like to caution against the conclusion that strain identity can therefore be neglected. On the contrary, strain specificity and multiplicity are important factors for disease progression and severity, and have significant leverage on treatment of two reasons. First, there are several mechanisms by which multiple strain infections may be relevant that could not be investigated here and that are not captured by metabolic responses.^6^ Second, our results are based on only two strains of a genetically highly variable parasite. The relevance of these findings have yet to be fully elucidated, but might well play a role for a deeper understanding of host-parasite interactions, personalized healthcare, and the monitoring of disease control interventions.

## Figures and Tables

**Table 1:** Host survival postinfection, total parasitemia, and decrease of host body condition (mean ± SD) over time in the different *Trypansoma brucei brucei* infection treatments in mice

	Infection treatment				
	Control	R_7_	R_7_G_5_	R_7_G_7_	G_7_
Survival	> 525	99.1 ± 10.4	103.1 ± 17.6	100.0 ± 16.9	100.0 ± 12.0
log_10_ (parasitemia)	0 ± 0	9.50 ± 0.08	9.37 ± 0.09	9.43 ± 0.08	9.42 ± 0.08
Anemia	−0.2 ± 17.5	27.7 ± 10.0**	26.3 ± 12.8*	33.3 ± 7.2**	31.9 ± 16.4*
Thrombocytopenia	−7.6 ± 9.9	84.8 ± 1.3***	80.1 ± 5.0**	84.5 ± 5.0**	86.4 ± 3.0***

Survival = survival after infection in hours (experiment stopped after 525 hours); parasitemia = total parasite density per mL of mouse blood on day 4 postinfection (*red* and *green* combined in mixed infections); anemia = % decrease in erythrocyte counts from the start (average of days 0 and 1 postinfection) to the final measurement (day 4); thrombocytopenia = % decrease in thrombocyte counts from the start (average of days 0 and 1 postinfection) to the final measurement (day 4). Asterisks indicate significance of paired *t* tests on differences between parameter start values vs. values on day 4, *, *P* < 0.05; **, *P* < 0.01; ***, *P* < 0.001.

**Table 2:** Goodness of fit (R^2^*X*), predictivity (Q^2^*Y*), and coefficient values of orthogonal signal corrected-projection to latent structure-discriminant analysis (O-PLS-DA) models derived from the comparison of infected and control mice on days 3 and 4 postinfection in this study compared with two previous studies using a different *Trypansoma brucei brucei* strain,^21^ and a *Plasmodium berghei* infection^27^ to ascertain specificity of metabolite signature*

Parasite	*T. b. brucei* strains STIB777AE-G1 and STIB246BA-R1								*T. b. brucei* strain GVR 35	*P. berghei*
Treatment†	G_7_		R_7_		R_7_G_5_		R_7_G_7_		–	–
Day postinfection	3	4	3	4	3	4	3	4	21	4
R^2^*X*	–	0.78	0.60	0.80	0.60	0.83	0.44	0.75	0.90	0.35
Q^2^*Y*	0.30	0.87	0.68	0.95	0.62	0.95	0.70	0.95	0.86	0.57
**Phenylpyruvate**		0.89	0.77	0.82	0.81	0.78	0.87	0.92		
**Guanidoacetate**		−0.92	−0.84	−0.96	−0.91	−0.97	−0.88	−0.94		
**4-hydroxyphenylpyruvate**		0.83		0.81	0.81	0.78	0.84	0.92		
Hippurate		−0.89		−0.86		−0.86	−0.83	−0.94	−0.71	
4-hydroxyphenylacetate		0.89		0.93		0.81	0.82	0.96	0.73	
**1-methylnicotinamide**		−0.92		−0.86		−0.83		−0.90		
**2-oxoglutarate**		−0.84								
**2-oxoisocaproate**		−0.91		−0.88		−0.80		−0.91		
3-methyl-2-oxovalerate						0.77	0.80	0.97	0.84	
D-3-hydroxybutyrate								0.94	0.74	
3-carboxy-2-methyl-3-oxopropanamine						0.81		0.96	0.75	
2-oxoisovalerate								0.84	0.69	
Lactate								0.86	0.66	
**Pyruvate**								0.93		
Trimethylamine									0.51	
Tryptophan									0.78	
Dimethylamine										0.58
Trimethylamine-*N*-oxide										−0.58
Phenylacetylglycine										0.66
Taurine										−0.58
Pipecolic acid										0.78

* Metabolites significantly contributing to the discrimination between non-infected and *T. b. brucei-*infected mice in O-PLS-DA models derived from urinary spectra in this study are highlighted using bold-type font. Positive values indicate higher concentrations of the metabolite in the infected mice and vice versa.

† G_7_ = infection with 10^7^ parasites of strain STIB777AE-G1; R_7_ = infection with 10^7^ parasites of strain STIB246BA-R1; R_7_G_5_ = infection with 10^7^ parasites of strain STIB246BA-R1 and 10^5^ parasites of strain STIB777AE-G1; R_7_G_7_ = infection with 10^7^ parasites of strain STIB246BA-R1 and 10^7^ parasites of strain STIB777AE-G1.
